# *Mycobacterium tuberculosis* Infection and Innate Responses in a New Model of Lung Alveolar Macrophages

**DOI:** 10.3389/fimmu.2018.00438

**Published:** 2018-03-12

**Authors:** Minjeong Woo, Connor Wood, Doyoon Kwon, Kyu-Ho Paul Park, György Fejer, Vincent Delorme

**Affiliations:** ^1^Tuberculosis Research Laboratory, Institut Pasteur Korea, Seongnam, South Korea; ^2^School of Biomedical and Healthcare Sciences, Peninsula Schools of Medicine and Dentistry, Plymouth University, Plymouth, United Kingdom; ^3^Applied Molecular Virology, Institut Pasteur Korea, Seongnam, South Korea

**Keywords:** Max Planck Institute cells, alveolar macrophages, *Mycobacterium tuberculosis*, innate response, cytokine secretion, autophagy, foamy macrophage

## Abstract

Lung alveolar macrophages (AMs) are in the first line of immune defense against respiratory pathogens and play key roles in the pathogenesis of *Mycobacterium tuberculosis* (*Mtb*) in humans. Nevertheless, AMs are available only in limited amounts for *in vitro* studies, which hamper the detailed molecular understanding of host-*Mtb* interactions in these macrophages. The recent establishment of the self-renewing and primary Max Planck Institute (MPI) cells, functionally very close to lung AMs, opens unique opportunities for *in vitro* studies of host-pathogen interactions in respiratory diseases. Here, we investigated the suitability of MPI cells as a host cell system for *Mtb* infection. Bacterial, cellular, and innate immune features of MPI cells infected with *Mtb* were characterized. Live bacteria were readily internalized and efficiently replicated in MPI cells, similarly to primary murine macrophages and other cell lines. MPI cells were also suitable for the determination of anti-tuberculosis (TB) drug activity. The primary innate immune response of MPI cells to live *Mtb* showed significantly higher and earlier induction of the pro-inflammatory cytokines TNFα, interleukin 6 (IL-6), IL-1α, and IL-1β, as compared to stimulation with heat-killed (HK) bacteria. MPI cells previously showed a lack of induction of the anti-inflammatory cytokine IL-10 to a wide range of stimuli, including HK *Mtb*. By contrast, we show here that live *Mtb* is able to induce significant amounts of IL-10 in MPI cells. Autophagy experiments using light chain 3B immunostaining, as well as LysoTracker labeling of acidic vacuoles, demonstrated that MPI cells efficiently control killed *Mtb* by elimination through phagolysosomes. MPI cells were also able to accumulate lipid droplets in their cytoplasm following exposure to lipoproteins. Collectively, this study establishes the MPI cells as a relevant, versatile host cell model for TB research, allowing a deeper understanding of AMs functions in this pathology.

## Introduction

Tuberculosis (TB) is a mycobacterial infection generally affecting the lungs. This ancestral disease, presents worldwide, affected around 10 million new patients and killed an estimated 1.8 million people in 2015 (1), despite the existence of a vaccine and an effective chemotherapy. The emergence of drug-resistant strains of *Mycobacterium tuberculosis* (*Mtb*), the main causative agent in humans, is an additional concern as it could further increase the risks of treatment failure. Host infection occurs when aerosolized, live bacteria reach the lower tract of the respiratory system and successfully evade innate immunity, usually resulting in the formation of a granuloma (2, 3). Resident lung alveolar macrophages (AMs) are among the first immune cells to encounter invading mycobacteria and the outcome of TB disease may be decided by the response of these macrophages to the incoming threat (4). The responses of AMs to pathogens, including *Mtb*, are very specific and distinct from other macrophages (5). Furthermore, entry into macrophages has been linked with metabolic changes in mycobacteria, increasing their resistance to antibiotics through the activation of efflux (6). Understanding how *Mtb* and AMs interact with each other is thus very important, but the difficulty in obtaining AMs in large quantity and in sufficient purity is a serious limiting factor. Depending on their origin, development, and environmental conditions, macrophages have distinct biological properties and significant functional differences exist among various macrophage populations. Previously, all tissue macrophages, including AMs, were believed to be bone marrow-derived cells with a limited life span. Recent studies, however, demonstrated that most tissue-resident macrophages, including AMs, are self-renewing cells of embryonic origin (7, 8). The unique characteristics of AMs are tailored by the special respiratory microenvironment, where granulocyte macrophage colony-stimulating factor (GM-CSF) drives the differentiation of AMs from embryonic macrophage precursors and sustains AM functions (7, 8).

Recently, a novel cellular model of embryonic derived, self-renewing tissue-resident macrophages [Max Planck Institute (MPI) cells] has been described (9). These GM-CSF dependent, primary cells represent an excellent model to study AM functions (9–11) but, in contrast to the scarcely available AMs, MPI cells can be obtained in virtually unlimited amounts. These very important properties could allow the use of these macrophages as a platform for high-throughput screening with drugs against *Mtb* and more generally, as a powerful tool for host-pathogen interaction studies in TB. Immortalized cell lines are routinely used due to their availability in large scale, but they often originate from tumors and/or were obtained through multiple passages; thus, their genetic background is not well defined and their phenotype can vary between lots. As such, they may not always be appropriate models to understand tissue-specific cellular functions (12, 13), or to correctly summarize critical interactions with pathogens, as reported in the case of *Leishmania* (14), adeno-associated virus (15), and *Mtb* (16, 17). In this context, the large-scale availability of MPI cells as a primary cellular model mimicking lung AMs could open new prospects in the understanding of pulmonary diseases, notably those involving complex host-pathogen interactions like TB.

Nevertheless, interactions of live *Mtb* with MPI cells have not been characterized so far. We report here that MPI cells constitute a suitable host cell system to study *Mtb* infection *in vitro*. This pathogen indeed enters and replicates in MPI cells in similar patterns as compared to other primary and non-primary cells. The innate cytokine response of MPI cells to live and HK *Mtb* was found to be characteristically different. Accordingly, MPI cells were able to target dead bacteria for phagolysosomal degradation. Altogether, our data show that MPI cells represent a particularly attractive and valuable tool for TB research.

## Materials and Methods

### Bacterial Culture

*Mycobacterium tuberculosis* strain H37Rv (ATCC27294) constitutively expressing the green fluorescent protein (GFP) (18), referred to as H37Rv-GFP, was grown in 7H9 broth (Invitrogen) supplemented with 10% Albumin-Dextrose-Saline, 0.05% Tween 80 (Sigma-Aldrich), 0.5% glycerol (Invitrogen), and 50 µg/mL hygromycin B (Invitrogen). Bacteria were grown for 14 days at 37°C, 5% CO_2_ in ventilated Erlenmeyer flasks without shaking, with dilution at OD_600 nm_ = 0.1 using fresh medium once a week. Bacteria were further grown at 37°C for 2 days with shaking at 200 rpm, harvested by centrifugation at 3,500 × *g* for 10 min and washed twice with phosphate buffered saline (PBS, Welgene) prior to infection. This protocol allowed us to collect well-individualized bacteria, without clumps.

### Cell Culture

Cells were grown at 37°C, 5% CO_2_ in RPMI 1640 medium (Welgene) supplemented with 10% heat-inactivated fetal bovine serum (FBS, Gibco), referred to as RPMI-FBS medium. Raw 264.7 murine macrophages were passaged every 2 or 3 days (70% confluence) and used between passages 2 and 9. THP-1 human macrophages were passaged once a week and differentiated in macrophages by 4 days exposure to 50 ng/mL phorbol 12-myristate 13-acetate (PMA, Sigma-Aldrich). Murine monocytes were obtained by flushing femurs of 6- to 8-week-old Balb/c mice (Orient Bio) with RPMI-FBS. Monocytes were differentiated into bone marrow-derived macrophages (BMDM) by exposure to 40 ng/mL recombinant murine macrophage colony-stimulating factor (M-CSF, Miltenyi Biotec) for 7 days. A seed stock of MPI cells established and characterized earlier (9) was used to generate all the MPI cells for this study. Cells from this stock were propagated in RPMI-FBS supplemented with 40 ng/mL recombinant GM-CSF (Miltenyi Biotec) to quickly increase cell density. MPI cells were collected and diluted at a concentration of 5 × 10^5^ cells/mL twice a week, for 3 weeks. Working stocks were prepared from the cells obtained after 6 passages (P6), by freezing aliquots of 10^7^ cells in 1 mL of FBS containing dimethyl sulfoxide (DMSO) (90:10, v/v) in liquid nitrogen. For each working stock, cells were thawed, washed, and grown in 20 mL of RPMI-FBS and 40 ng/mL of GM-CSF. MPI cells were further amplified in RPMI-FBS and 10 ng/mL GM-CSF and passaged twice a week at a concentration of 2 × 10^5^ cells/mL. Cells from passages 8 (P8) to 70 (P70) were used.

### *Mtb* Infection with Plated Cells for Microscopy Experiments

The day preceding *Mtb* infection, cells were treated with 1× Versene (Gibco) for 10 min at 37°C, gently detached using a cell-scraper, centrifuged at 300 × *g* for 5 min and resuspended in fresh RPMI-FBS medium. In the case of MPI cells, culture supernatants were also centrifuged to recover non-attached cells, which were pooled together with the scraped cells. Cells were enumerated using a Thoma cell counting chamber and suspensions at 5 × 10^5^ cells/mL were prepared. The medium was supplemented with 40 ng/mL M-CSF for BMDM or with 50 ng/mL PMA for THP-1 cells. First, 50 μL/well of the cell suspensions were distributed in 384-well plates (Greiner, μClear) to reach 25,000 cells/well. Plates were then incubated overnight at 37°C, 5% CO_2_ prior to infection, to allow the cells to attach at the bottom of the well. The day of the infection, pellets of freshly PBS-washed bacteria were resuspended in RPMI-FBS at the required concentration to yield the desired multiplicity of infection (MOI). For this, bacterial stocks were titrated on 7H11 agar beforehand, to determine the relationship between optical density and bacterial concentration. In our hands, suspensions of well-dispersed bacteria at OD_600 nm_ = 1 were reproducibly titrated at 1–3 × 10^8^ bacteria/mL. The medium was removed from the plate and 50 μL/well of bacterial suspension were added. For MPI cells, plates were also centrifuged at 300 × *g* for 2 min prior to medium removal, to sediment non-adherent cells.

Plates were further incubated for 4 h at 37°C, 5% CO_2_, then centrifuged at 300 × *g* for 2 min. The medium was removed and 50 µL/well of fresh RPMI-FBS supplemented with 5 µM Hoechst 33342 (Sigma-Aldrich) was added. Plates were incubated 20 min at 37°C, 5% CO_2_ before imaging using fluorescence microscopy (Operetta, Perkin Elmer). Of importance here, when comparing different cell lines together, the same bacterial suspension was used for infection, in order to limit variations and allow more relevant comparisons.

### Quantification of Cytokine Secretion

Heat-killed (HK) bacteria were prepared by boiling washed bacteria resuspended in PBS (OD_600 nm_ = 1) for 30 min at 95°C. Aliquots were prepared and frozen at −80°C until subsequent use. MPI cells or BMDM were gathered and enumerated as indicated above to prepare suspensions at 5 × 10^5^ cells/mL and plated overnight in 24-well plates (Costar, 1 mL/well) at 37°C, 5% CO_2_. The plates were centrifuged at 300 × *g* for 2 min, the medium removed and 1 mL/well of bacterial suspension or lipopolysaccharide (LPS) at the appropriate concentration was added. At the corresponding time points, the plates were centrifuged and the supernatants were recovered and sterile-filtered through a 0.22 µm PVDF syringe-driven filter (Millex, Millipore). Filtered supernatants were stored at −80°C prior to quantification. Cytokines were titrated by ELISA, using antibody pairs directed against interleukin 6 (IL-6), TNF-α, IL-1β, IL-10 (ThemoFisher Scientific), and IL-1α (Peprotech), following manufacturers’ recommendations. All data points were measured in triplicates, and sample concentrations were calculated against a standard curve generated with recombinant cytokines (ThermoFisher Scientific). Samples were tested at different dilutions, ranging from non-diluted to 50-fold dilutions, to ensure accurate calculation of the cytokine concentrations. All experiments were done in triplicates.

### Batch Infection and Isoniazid Activity

Isoniazid (INH, Sigma-Aldrich) stock solutions were prepared at 1 mM in DMSO (Sigma-Aldrich) and kept at −20°C. Cells were gathered and enumerated as indicated above, to prepare suspensions at 10^6^ cells/mL. Pellets of freshly PBS-washed bacteria were resuspended in RPMI-FBS at the required concentrations to yield the desired MOI. One volume of bacterial suspension was mixed with one volume of cell and the mixture was incubated for 2 h at 37°C with mild shaking (100 rpm). Infected cells were washed twice with fresh medium and plated at 25,000 cells/well in 384-well plates containing twofold dilutions of isoniazid in RPMI-FBS (the dose–response experiments were performed in triplicates). Where required, the final volume in each well was adjusted to 50 µL using RPMI-FBS and final concentrations of DMSO were kept below 1%. BMDM were incubated with 20 ng/mL M-CSF and THP-1 cells with 50 ng/mL PMA. After 5 days incubation at 37°C, 5% CO_2_, 10 µL/well of a 30 µM Hoechst 33342 solution in PBS was added (final concentration 5 µM) and plates were incubated for 20 min at 37°C, 5% CO_2_ before imaging using fluorescence microscopy.

### Autophagy Assessment

Cells were plated overnight, as described above, in 384-well plates containing either DMSO (1% final) or chloroquine (Invitrogen, 50 µM final concentration). Plated cells were infected for 2 h at 37°C, 5% CO_2_, then fixed by treatment with 50 μL/well of 10% neutral buffered formalin (Sigma-Aldrich) for 30 min at room temperature. Cells were washed twice with PBS and permeabilized using 0.2% Triton X-100 (Sigma-Aldrich) in PBS for 15 min at room temperature. The medium was removed and a solution of light chain 3B (LC3B)-directed rabbit polyclonal antibodies (Invitrogen) in PBS (1 µg/mL) was added (50 µL/well). Plates were incubated overnight at 4°C and washed three times with PBS. A solution of goat anti-rabbit IgG (H + L) secondary antibodies conjugated with Alexa Fluor 568 probe (Invitrogen) was prepared at 5 µg/mL in PBS and incubated with the cells (50 µL/well) for 1 h at room temperature. Cells were washed three times with PBS and finally stained with 5 µM Hoechst 33342 solution in PBS (50 µL/well) for 30 min before imaging using fluorescence microscopy.

### LysoTracker Staining

Cells were plated in 384-well plates and infected for 2 h, as described above. For infection with formalin-killed bacteria, pellets of bacterial culture were resuspended in formalin (OD_600 nm_ = 1) and incubated overnight at room temperature. Formalin-killed bacteria were washed twice with PBS, resuspended in RPMI-FBS at the required MOI, and used similarly to live bacteria. After infection, plates were centrifuged at 300 × *g* for 2 min, the medium was removed, and cells were incubated for 2 h with 50 µL/well of 0.5 µM LysoTracker Deep Red (Invitrogen) in RPMI-FBS. Cells were stained by addition of 10 µL/well of 30 µM Hoechst 33342 in PBS (final concentration 5 µM) for 30 min before imaging using fluorescence microscopy.

### Preparation of Lipid-Loaded Cells

Cells were plated overnight as described above and medium was replaced with 50 µL/well of fresh RPMI-FBS supplemented with very low-density lipoproteins (VLDL, Merck Millipore), to reach a final concentration of 140 µg triacylglycerol (TAG) per mL of medium (VLDL were usually received at a concentration of 9–10 mg TAG/mL). Plates were incubated up to 6 days at 37°C, 5% CO_2_ and regularly checked under a light microscope. Accumulation of lipid droplets was usually observed 2–4 days after exposure to VLDL. At this stage, plates were centrifuged at 300 × *g* for 2 min, the medium was removed, and cells were fixed by treatment with formalin (50 µL/well) for 30 min at room temperature. Cells were washed twice with PBS and stained with 5 µM Hoechst 33342 in PBS (50 µL/well) for 30 min. Cells were washed again three times and a 1/100 dilution in PBS of LipidTOX Deep Red neutral lipid stain (Invitrogen) was added (50 µL/well). Cells were incubated at room temperature for 30 min before imaging using fluorescence microscopy.

### Image Acquisition and Analysis

Nuclei channel (Hoechst 33342) was obtained using excitation (Ex): 405 nm and emission (Em): 450 nm. Bacteria channel (GFP) was obtained using Ex: 488 nm; Em: 520 nm. LC3B channel (Alexa Fluor 568) was obtained using Ex: 561 nm; Em: 600 nm. Finally, both lysosome and lipid channels (LysoTracker Deep Red and LipidTOX Deep Red) were obtained using Ex: 640 nm; Em: 690 nm. Color images were obtained through the software Harmony v3.5 (Perkin Elmer). At least three fields were imaged for each well. For each field, objects (cells, bacteria, and vacuoles) were segmented from individual channels using in-house algorithms, all based on commonly available procedures like watershed and threshold-based algorithms. Detected cells were counted and split in sub-groups [infected/non-infected (NI) bystander cells and/or stained/unstained cells, as appropriate]. Ratios of infected/stained cells were calculated as the number of infected/stained cells divided by the total number of cells. The number of pixels (px) found to be positive for GFP signal in the bacterial channel was referred to as the bacterial area, expressed in px. For each infected/stained cell, the area of intracellular bacteria/stain (in px) was recorded to allow a calculation of the average area of intracellular bacteria/stain for all the cells in the group, referred to as the bacterial/stain area per cell (expressed in px). Values for each field were averaged to give a value per well. Values per well were considered to be individual replicates (*n*) and pooled together to plot the graphs and determine SD.

### Statistical Analyses

**Figure 1:**
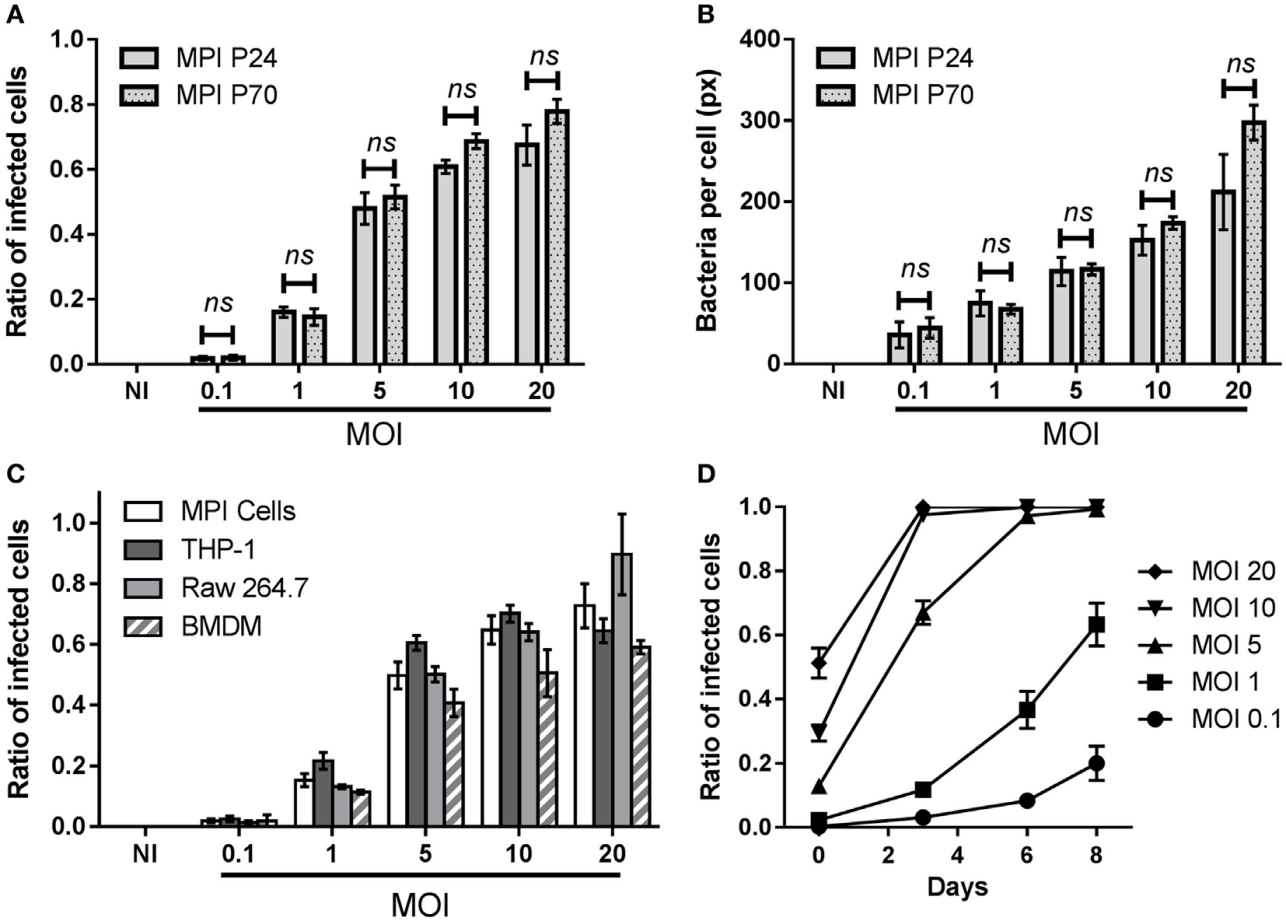
Infection patterns of Max Planck Institute (MPI) cells with *Mycobacterium tuberculosis* (*Mtb*). Comparison of the phagocytic capacity of MPI cells obtained after 24 (P24) or 70 (P70) passages, 4 h post-infection with *Mtb*. **(A)** Ratio of infected cells obtained at various multiplicity of infection (MOI). **(B)** Average area of bacteria per infected cells (in pixels). Values are mean ± SD (*n* = 4) for this representative infection experiment. At each MOI, differences between P24 and P70 were found to be non-significant (ns) by two-way ANOVA. **(C)** Comparison of the phagocytic capacity of MPI cells (P28), THP-1 cells, Raw 264.7, and bone marrow-derived macrophages (BMDM). Cells were plated overnight and incubated for 4 h with aliquots of bacterial suspension from the same batch. Values are mean ± SD (*n* = 4) for this representative infection experiment. **(D)** Kinetics of H37Rv-green fluorescent protein replication in MPI cells (P42) for a representative batch infection (100 rpm, 2 h), using different MOI. At each time point, three wells were used for the quantification of the ratio of infected cells using fluorescence microscopy. Values are plotted as mean ± SD.

**Figure 2:**
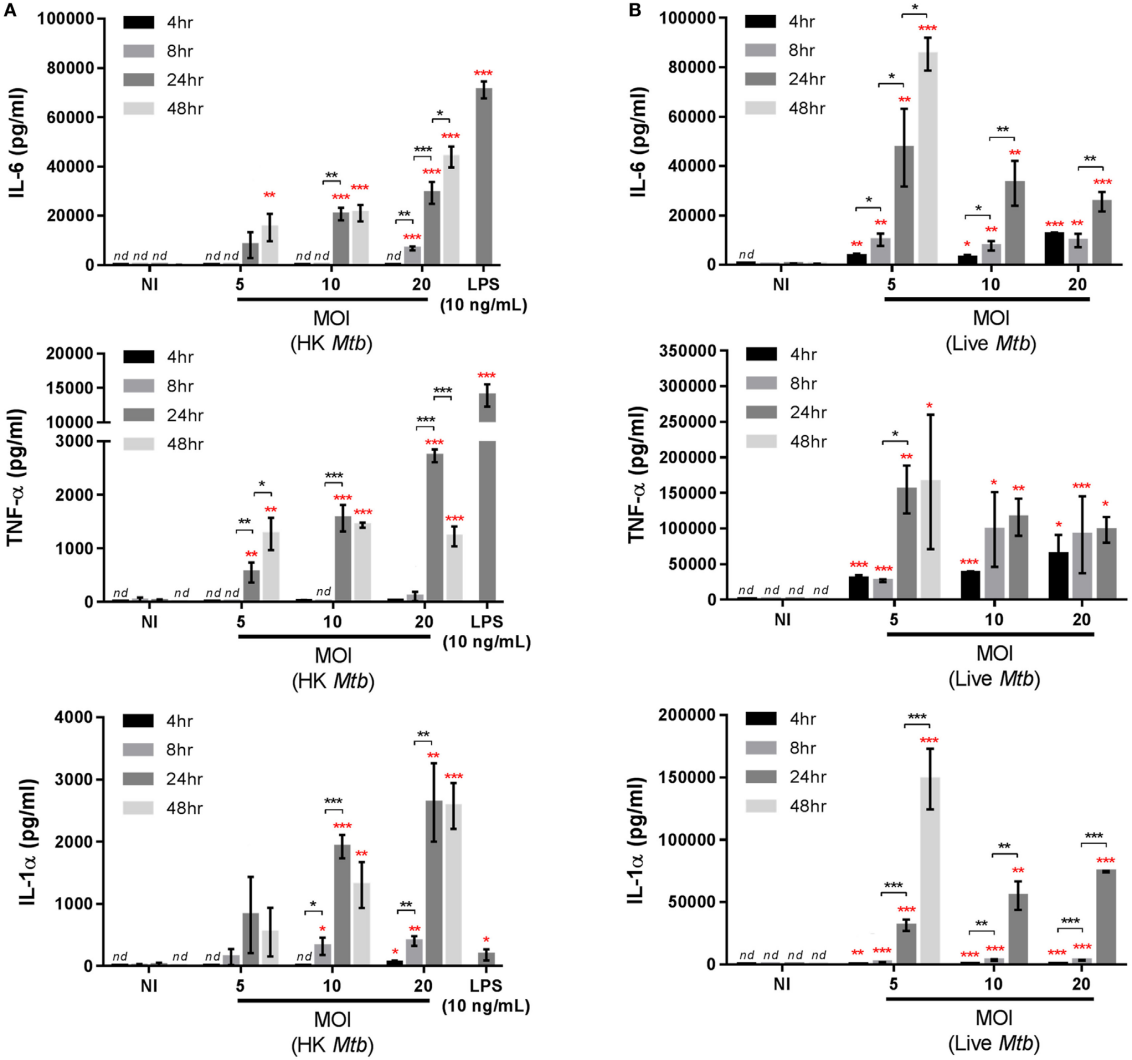

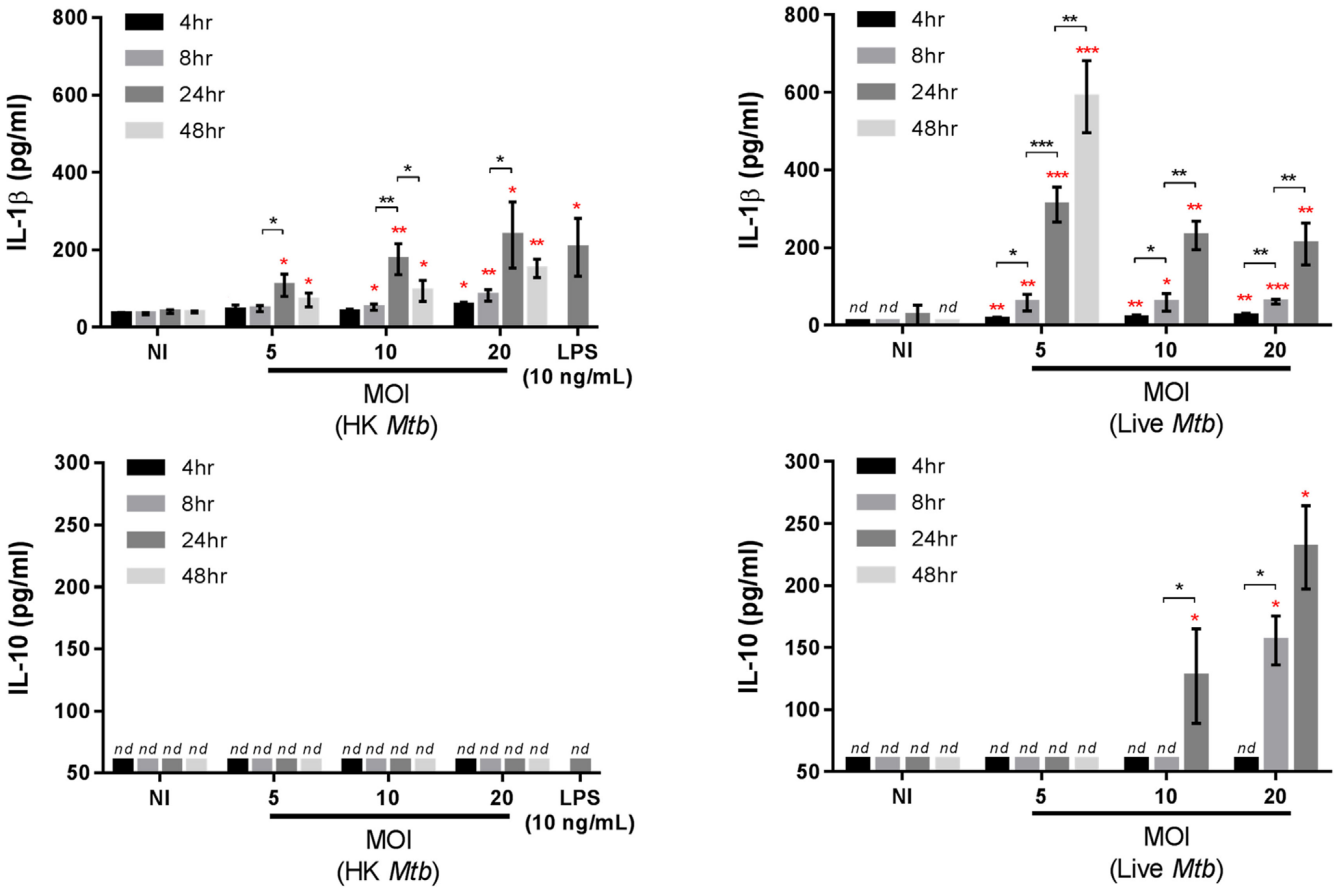
Kinetics of cytokine secretion in Max Planck Institute (MPI) cells. Production of interleukin 6 (IL-6), TNF-α, IL-1α, IL-1β, and IL-10 by MPI cells (P32) in response to **(A)** heat-killed (HK) *Mycobacterium tuberculosis* (*Mtb*) or 10 ng/mL lipopolysaccharide (LPS) or **(B)** live *Mtb* infection challenge. *Mtb* was used at different multiplicity of infection (MOI) and cytokines were quantified at 24 h post-treatment for LPS, 4, 8, 24, and 48 h post-infection with HK bacteria and 4, 8, 24, and 48 h (MOI 5) or 4, 8, and 24 h (MOI 10 and 20) post-infection with live bacteria. Data are representative of three independent experiments and expressed as mean ± SD for three biological replicates. Statistical significance between time points (black asterisks) or between stimulated and non-stimulated cells at corresponding time points (red asterisks) were determined by Prism software using the unpaired Student’s *t*-test. *, *p*-value < 0.05; **, *p*-value < 0.01; ***, *p*-value < 0.001. *nd*, not detectable. Nothing was indicated when the differences were found to be non-significant.

**Figure 3:**
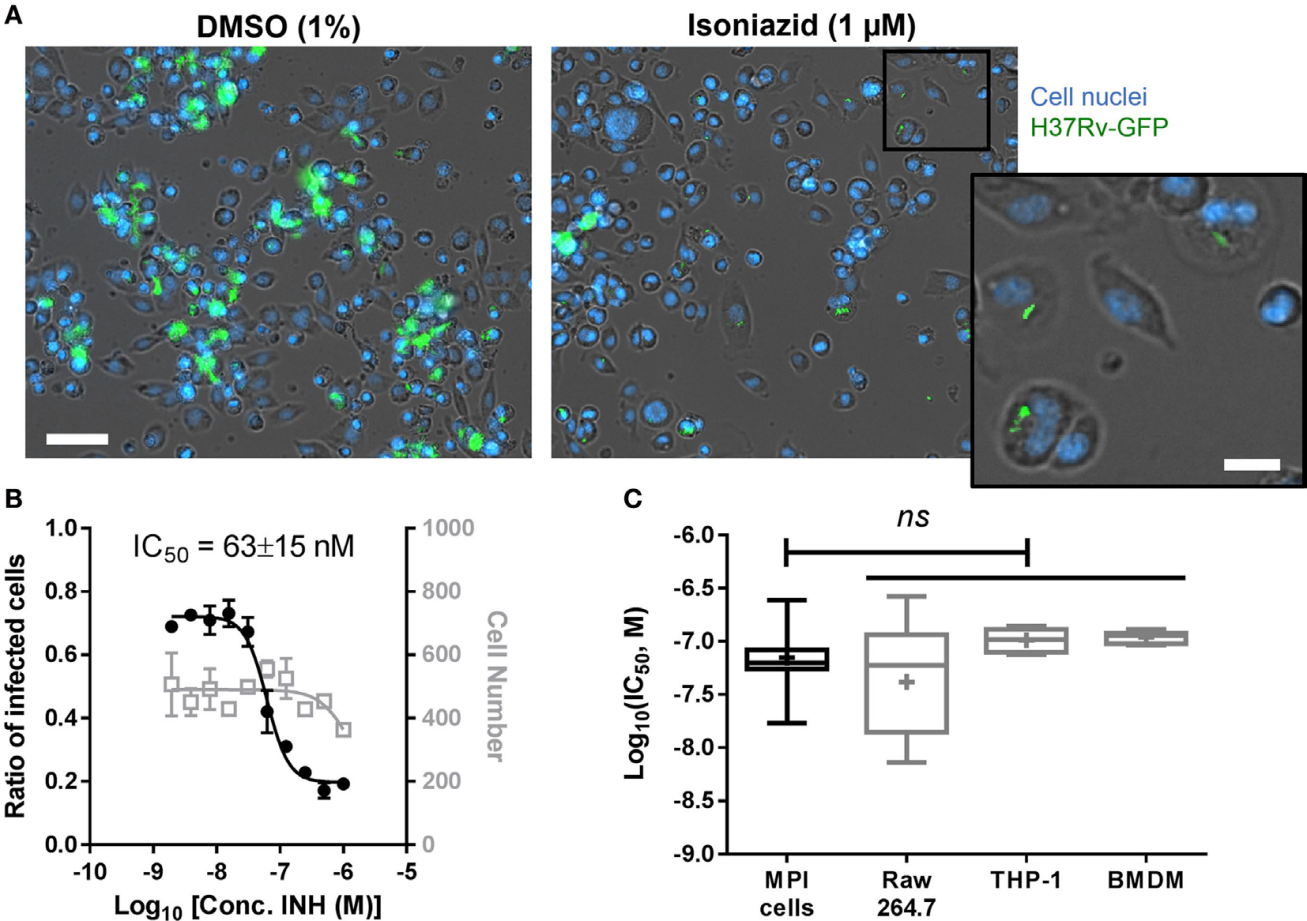
**(A)** Treatment of *Mycobacterium tuberculosis*-infected Max Planck Institute (MPI) cells with the anti-tuberculosis drug isoniazid. Representative pictures of MPI cells (P32, blue) treated with dimethyl sulfoxide (DMSO) or isoniazid (INH) for 5 days after batch infection (100 rpm, 2 h) with H37Rv-green fluorescent protein (GFP) (green) at a multiplicity of infection (MOI) of 5. Cell nuclei were stained with Hoechst 33342 (blue). Scale bar: 50 µm. Inset from isoniazid treated cells shows magnified view of persistent bacterial infection in intact cells. Scale bar: 15 µm. **(B)** Dose–response of isoniazid against H37Rv-GFP replicating in MPI cells (P18, MOI 10). The ratio of infected cells is shown with black, closed circles; the number of cell is shown with gray, open squares. Fitting were performed by Prism software using a sigmoidal dose–response model and used to determine the concentration of isoniazid required to inhibit 50% of the bacterial colonization process (IC_50_). Data are mean ± SD for three replicated dose–response. **(C)** Comparison of isoniazid IC_50_ values (expressed in log scale) for different cell types. The number of infection experiments pooled to build the box plots was 5 for MPI cells and Raw 264.7 cells, 3 for THP-1 cells and bone marrow-derived macrophages (BMDM). Box plots are showing the max and min values (brackets), 75th and 25th percentiles (edges of the box), the median value (horizontal bar), and the mean value (+). Statistical differences between MPI cells and other cells were determined as non-significant (*ns*) using a one-way ANOVA with Dunnett’s multiple comparisons test.

**Figure 4:**
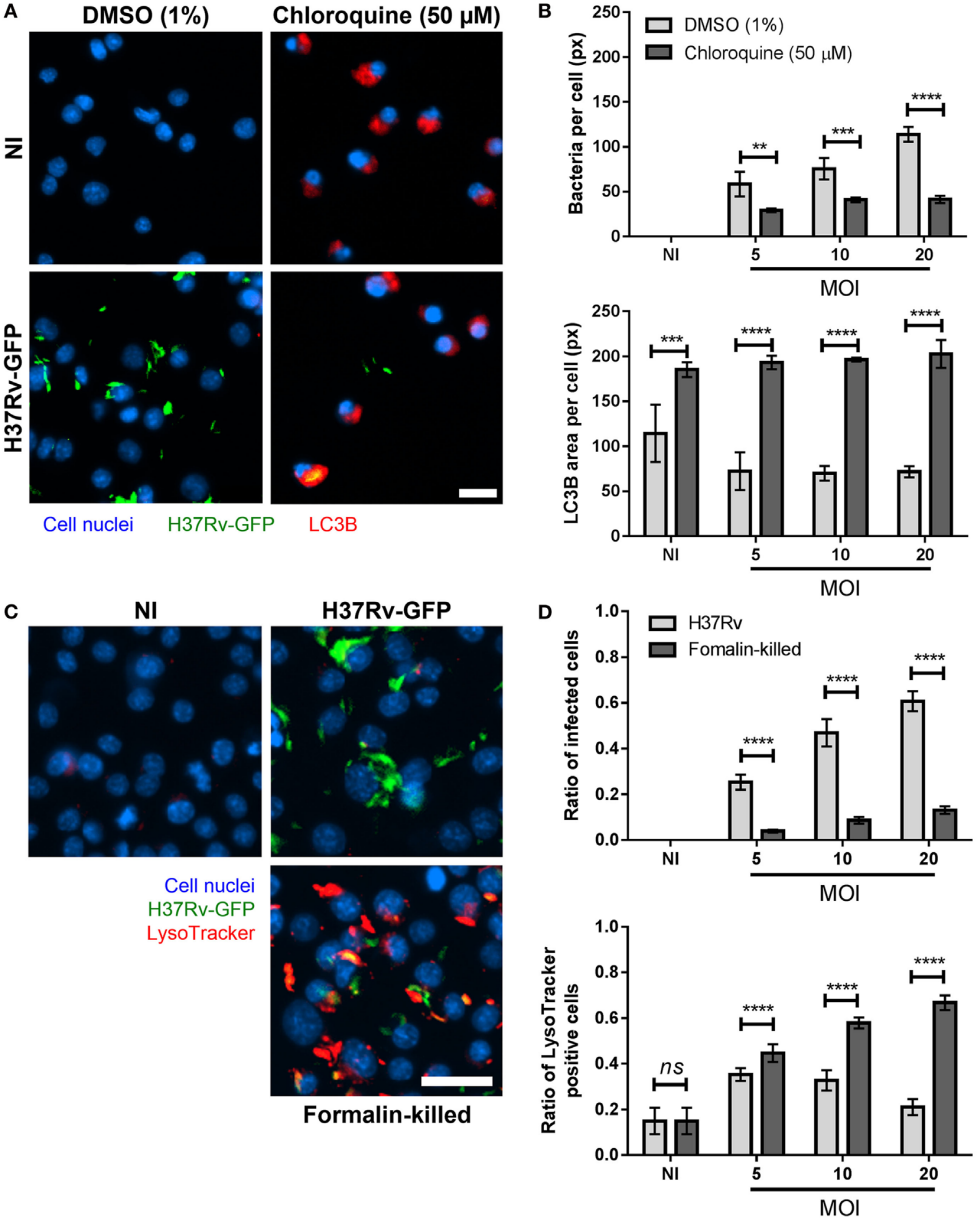
Light chain 3B (LC3B) positive and acidic vacuoles labeling in *Mycobacterium tuberculosis*-infected Max Planck Institute (MPI) cells. **(A)** Representative pictures of dimethyl sulfoxide (DMSO) and chloroquine-treated MPI cells (P54, blue), non-infected (NI) and infected with H37Rv-green fluorescent protein (GFP) (green) at a multiplicity of infection (MOI) of 10 and labeled with LC3B antibody (red). Scale bar: 15 µm. **(B)** Comparison of the average area of bacteria per infected cells (top) and the average area of LC3B puncta per labeled cells (bottom), between DMSO and chloroquine treated MPI cells infected at different MOI. Values are mean ± SD (*n* = 3). **(C)** Representative pictures of MPI cells (P38, blue), NI and infected with live or formalin-killed H37Rv-GFP (green) at a MOI of 10 and labeled with LysoTracker Deep Red dye (red). Scale bar: 25 µm. **(D)** Average ratio of infected cells and ratio of LysoTracker positive cells obtained with MPI cells NI or infected with live or formalin-killed H37Rv-GFP bacteria. Values are mean ± SD from nine biological replicates. Statistical significance was determined by Prism software using two-way ANOVA. *ns*, non-significant; **, *p*-value < 0.005; ***, *p*-value < 0.0005; ****, *p*-value < 0.0001.

**Figure 5:**
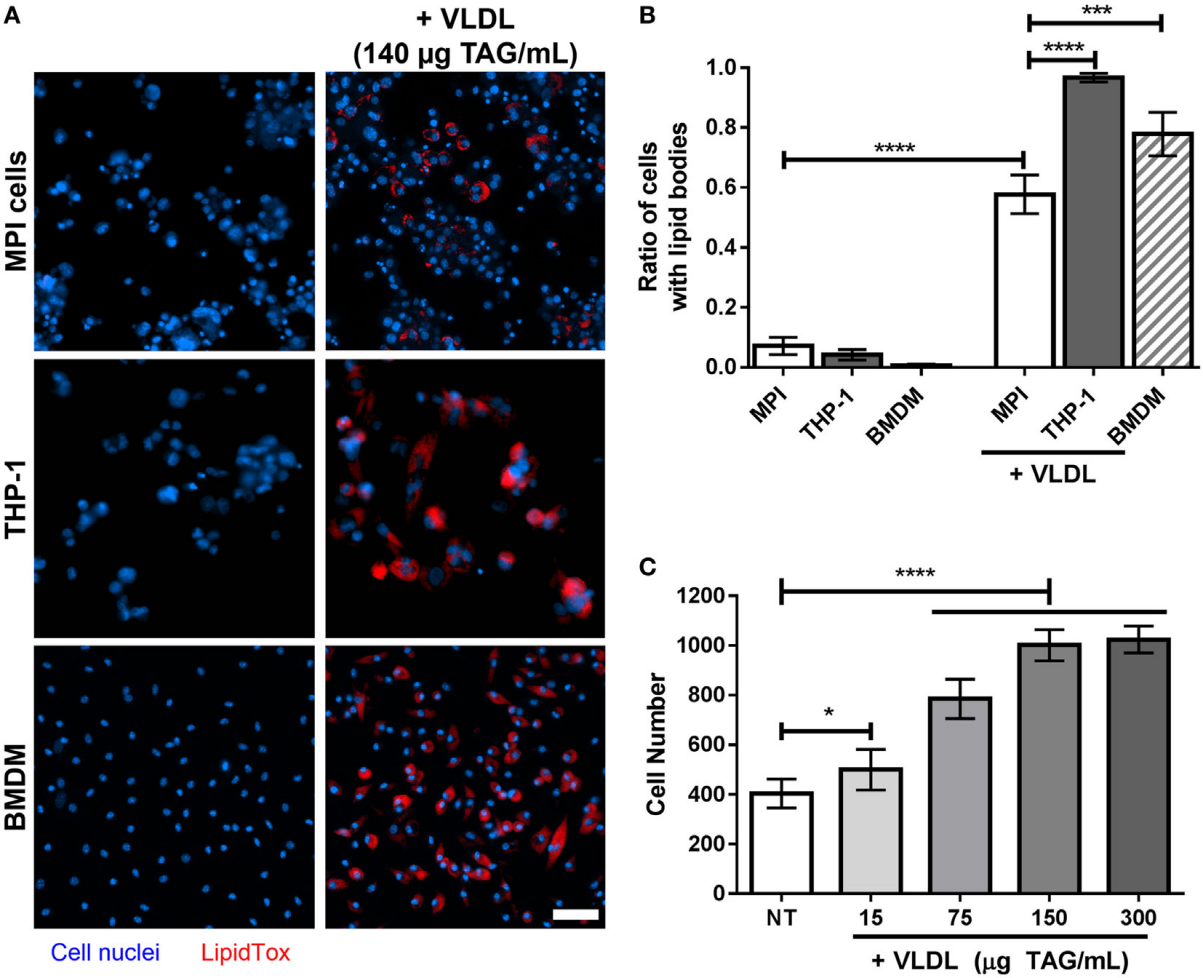
**(A)** Lipid droplets accumulation in cells treated with very low-density lipoproteins (VLDL). Representative pictures of naïve and VLDL-treated THP-1 cells, Max Planck Institute (MPI) cells (P20), or bone marrow-derived macrophages (BMDM) (blue). The VLDL concentration was adjusted to reach a final concentration of 140 µg triacylglycerol (TAG)/mL. Cells were stained with LipidTOX neutral lipid dye (red). Scale bar: 50 µm. **(B)** Comparison of the ratio of LipidTOX stained cells in the presence and absence of VLDL, for different cell types (*n* = 9). **(C)** Replication of MPI cells (P26) in the presence of various amounts of VLDL (expressed as final concentration of TAG/mL), after 4 days of incubation. Results are mean ± SD from nine biological replicates. Statistical significance compared to the non-treated (NT) sample was determined by Prism software using Student’s *t*-test. *, *p*-value < 0.05; ***, *p*-value < 0.0005; ****, *p*-value < 0.0001.

All experiments were repeated with at least three different batches of infected cells and showed similar results. Data shown are for a representative infection experiment, except for Figure 3C, where the number of pooled infections is indicated in the legend. For each infection experiment, samples were tested at least in triplicates. All graphs were built with Prism software v6.0 (GraphPad). The same software was used to calculate statistical significance of the differences between the indicated data points. A two-way ANOVA with Tukey’s multiple comparisons test was used for Figures 1 and 4, a one-way ANOVA with Dunnett’s multiple comparisons test was used for Figure 3C, and the unpaired Student’s *t*-test was used for Figures 2 and 5.

## Results

### Adjustment of Culture Conditions for *Mtb* Infection

Under the microscope, MPI cells displayed a homogenous, round shape (Figure S1 in Supplementary Material) that was consistent with all passages tested (up to P70). The cells grew both attached at the surface of the flasks and in suspension. Cells plated in 384-well plates were easily removed by washings with fresh medium, as shown in Figure S2A in Supplementary Material. This undesired loss of cells was, however, easily circumvented by a centrifugation step between each washing (2 min at 300 × *g* was found to be sufficient). Once fixed using formalin, the cells displayed higher adherence and centrifugation was no longer necessary (Figure S2B in Supplementary Material). It was thus possible to obtain a dense population of cells with minimal losses even for immunostaining experiment, requiring multiple washing steps. After plating and growth without GM-CSF, MPI cells quickly stopped multiplying and a steady number of cells could be observed in the plate for up to 1 week (Figure S2C in Supplementary Material).

### Phagocytic Capacity of the MPI Cells and Its Stability for Live *Mtb*

We tested the capacity of plated MPI cells obtained after different passages to phagocytose live, fluorescent *Mtb* (H37Rv-GFP). This allowed us to compare the phagocytic capacity of low and high passage number cells, i.e., obtained below 30 or above 50 passages, respectively. As shown in Figure 1A and Figure S3 in Supplementary Material, MPI cells collected after passages 24 (P24) and 70 (P70) efficiently took up *Mtb* and displayed similar phagocytic patterns, with equivalent ratios of infected cells and average amount of bacteria per infected cells observed 4 h post-infection at all the different MOI tested. The same conclusion was drawn for the non-pathogenic mycobacteria *Mycobacterium smegmatis*, against which MPI cells collected at P12 and P52 showed a comparable internalization pattern (Figure S4 in Supplementary Material). This indicated that the capacity of the MPI cells to phagocytose live mycobacteria was efficient and stable even after numerous passages. Furthermore, we wanted to compare the phagocytic capacity of the MPI cells with that of other macrophages routinely used to study *Mtb* infection, namely the Raw 264.7 murine cell line, differentiated human THP-1 cells and BMDM. While it was difficult to infect exactly the same number of cells from each cell types, a similar trend was observed over three independent infection experiments, showing that all cell types had comparable phagocytic capacity against *Mtb* 4 h post-infection. A representative result is illustrated in Figure 1B (additional parameters shown in Figure S5 in Supplementary Material), obtained with MPI cells at P28.

Max Planck Institute cells (P42) were also successfully infected as suspended cells under mild shaking (batch infection), as shown in Figure 1C. Of note, as batch infections were carried out for only 2 h, the ratios of infected cells observed after infections were lower than those obtained with plated cells, for an equivalent MOI (Figure 1A). The kinetics of bacterial replication within plated MPI cells were followed for 8 days, showing an increasing number of GFP positive cells over time (Figure 1D). MPI cells were able to withstand low MOIs (1 or below) during this period, while MOIs of 10 and above resulted in a near complete destruction of the cells in less than 3 days.

### *Mtb*-Induced Cytokine Secretion Profiles in MPI Cells

The cytokine secretion profiles of MPI cells were characterized after infection with live or HK *Mtb*, as well as to LPS stimulation. Different MOIs and time points were assessed. Infection of MPI cells with HK bacteria (Figure 2A) resulted in the production of IL-6 and IL-1α from 24 h post-infection, in a MOI-dependent manner, while no IL-10 was detected (detection limit estimated to be ~60 pg/mL). Furthermore, HK *Mtb* also induced secretion of tumor necrosis factor alpha (TNF-α) and, in a lesser extent, IL-1β in correspondence with previous findings (9), which validates the phenotype of the MPI cells used in this study. Infection of MPI cells with live *Mtb*, however, led to a more robust cytokine production. As indicated in Figure 2B, there was an increase in IL-6, TNF-α, IL-1α, and IL-1β over time, for all MOIs tested. In contrast to HK *Mtb*, the induction of these cytokines started as early as 4 h post-infection and increased at 24 h. At a MOI of 5, the increase in cytokine secretion was sustained up to 48 h post-infection (of note, at MOI 10 and 20, the 48 h data point was not assessed due to an already advanced destruction of the cellular monolayer). Interestingly, higher MOIs did not induced stronger responses, indicating that optimal stimulations were already achieved at MOI 5. The levels of induced cytokines were moderately (IL-6) or dramatically (TNF-α, IL-1α) higher than those obtained with HK *Mtb* or LPS, both being strong stimulators of cytokine production in MPI cells (9). Importantly, IL-1β was also found in significant amounts in the supernatants, particularly at 24 and 48 h post-infection (310 and 590 pg/mL, respectively). Surprisingly, MPI cells challenged with live *Mtb* also produced detectable amounts of IL-10 at 24 h post-infection (120 and 230 pg/mL at MOI 10 and 20, respectively). A comparison between MPI cells and BMDM was also performed (Figure S6 in Supplementary Material), showing that 8 h after infection with live bacteria, MPI cells were secreting more TNF-α, comparable amounts of IL-6 and less IL-1β than BMDM, at all the MOI tested. Taken together, these data demonstrate that the MPI cells display a distinct and characteristic pro-inflammatory cytokine secretion profile in response to *Mtb* infection.

### Response to Anti-TB Drug Treatment

The prospect of conducting high-throughput screening using primary cells is often hampered by the elevated costs needed to obtain such cells in sufficient quantities, as well as the need for high homogeneity between batches to allow reproducible results. To assess the suitability of MPI cells for compound and drug screening in the context of TB, we investigated the effects of the first line anti-TB agent isoniazid on the intracellular replication of *Mtb* in MPI cells. As shown in Figure 3A, when treated with 1 µM isoniazid for 5 days, MPI cells displayed strongly reduced bacterial loads in their cytoplasm as compared to the control cells (1% DMSO). Persistent, presumably live bacteria were still seen after treatment, however, indicating the possibility for *Mtb* to survive for an extended period of time within MPI cells despite high concentrations of drug in the culture medium. Of note, a similar phenomenon is regularly observed with other cells routinely used in our laboratory for compound testing. Nevertheless, a dose–response effect was observed for isoniazid (Figure 3B), with a concentration required to inhibit 50% of the bacterial replication (IC_50_) estimated at 50–80 nM in these conditions. A plateau of efficacy was reached for isoniazid above 250–500 nM. Consistent IC_50_ values were measured for isoniazid using other cell types, namely Raw 264.7 cells, THP-1 cells, and BMDM (Figure 3C). The reproducibility of the IC_50_ values measured for MPI cells over five independent experiments was good (3.9% CV), better than that obtained with Raw 264.7 cells (7.6% CV). In summary, MPI cells were found to respond very similarly to other cell types following isoniazid treatment, both in terms of phenotype and measurable drug activity. While not shown here, additional dose–responses performed with rifampicin and moxifloxacin indicated similar conclusions.

### Autophagy and Phagosomal Acidification

Autophagy is a conserved mechanism allowing cells to degrade and recycle intracellular organelles by delivering them to lysosomes. Autophagy has also been described as a defense mechanism allowing macrophages to eradicate intracellular mycobacteria, despite the fact that they normally block the phagosome-lysosome fusion process (19–21). To assess the capacity of MPI cells to target intracellular pathogenic *Mtb* into autophagosomes, immunostaining experiments were performed using antibodies directed against the microtubule-associated protein 1A/1B-LC3B, described as a marker of autophagosomes (22). In the control condition (1% DMSO), both NI and infected MPI cells displayed few LC3B staining (Figures 4A,B), with less than 20% of the cells being positive for the immunostaining (Figure S7 in Supplementary Material). This indicated the successful escape of the virulent bacteria from autophagosome delivery. By contrast, in the presence of 50 µM chloroquine, a known inducer of autophagic vacuoles (23), around 90% of the cells were found to be positive for LC3B labeling (Figure S7 in Supplementary Material). In addition, infected cells displayed a strongly reduced bacterial load in their cytoplasm at all MOIs tested (41 px versus 114 px without chloroquine at MOI 20, *p*-value < 0.0001; Figure 4B). This suggested that live, virulent bacteria were rapidly eliminated within autophagolysosomes, in accordance with previous studies (19).

In a similar fashion, we studied the phagosomal acidification process within MPI cells after infection with *Mtb*. The LysoTracker dye was used to follow the appearance of acidic vacuoles in the cell cytoplasm following infection with *Mtb*. NI cells presented very few staining (Figure 4C). When infected with live *Mtb*, a slight increase in the number of intracellular acidic vacuoles was observed 2 h post-treatment, especially at the lowest MOI tested (Figure 4D; Figure S8 in Supplementary Material). This appearance of acidic vacuoles was, however, significantly higher when the bacteria were killed by exposure to formalin prior to infection (66% of positive cells against 21% for live bacteria at MOI 20, *p*-value < 0.0001; Figure 4D). This was concomitant with a reduction in the number of formalin-killed bacteria detected inside the cells (80 px versus 57 px with live bacteria at MOI 20, *p*-value < 0.0005; Figure S8 in Supplementary Material), resulting in a low ratio of infected cells but a high amount of LysoTracker positive cells and overall LysoTracker area, suggesting the successful targeting and destruction of dead bacteria within phagolysosomes (Figures 4C,D; Figure S8 in Supplementary Material). Taken together, these results show that MPI cells are successfully driving formalin-killed bacteria to phagolysosomes for degradation, while live *Mtb* manage to escape this process, in accordance with observations previously reported for BMDM (24). Of note, the non-primary Raw 264.7 and THP-1 cells are less suitable for experiments with LysoTracker, as they usually display non-specific background staining without infection (Figure S9 in Supplementary Material). This further highlights the relevance of the MPI cells as a versatile tool for host-pathogen interactions studies in TB.

### Lipid Accumulation

Lipid-loaded macrophages, or foamy macrophages, have an increasing significance in TB research given their potency to be used as a platform for the study of mycobacterial intracellular persistence and reactivation (25). In a recent study, a model using VLDL has been described to generate foamy macrophages *in vitro* from BMDM (26). We tested whether MPI cells could also be used for this purpose. A strong increase in lipid staining was observed for MPI cells after treatment with VLDL (58% of cells stained against 7% without VLDL treatment, *p*-value < 0.0001; Figures 5A,B). However, the ratio of MPI cells stained after 4 days of incubation was found less prominent than that observed with THP-1 cells and BMDM (corrected *p*-value < 0.0001). We also noticed that the density of MPI at the end of the incubation period was higher than expected, which was confirmed by exposure of the MPI cells to increasing doses of VLDL (Figure 5C). This indicated that some components within the lipoprotein suspension were able to induce the proliferation of the cells, an unexpected effect that needs to be considered when carrying out such experiments.

## Discussion

In this report, we present the first characterization of the lung AM model MPI cells in the context of *Mtb* infection. Our data indicate that these macrophages are well suited for infection experiments and can be used as a model to study the process of autophagy and phagosomal acidification in *Mtb*-infected cells. In particular, MPI cells displayed the ability to phagocytose *Mtb* and quickly eliminate formalin-killed bacteria through phagosome acidification, while live mycobacteria were successfully evading this pathway. Together, these data show that MPI cells are competent phagocytes of *Mtb* and that the mechanisms enabling the bacteria to evade destruction and multiply inside the phagosomes are functional in these cells.

Max Planck Institute cells have the capacity to grow as both attached and free-floating cells. While difficult to quantify, it also appears clearly throughout our experiments that the MPI cells had the tendency to aggregate and form clumps after *Mtb* infection, as visible in Figure 3A for the DMSO-treated cells. This might be linked with the active movement of these cells, able to detach and reattach, similarly to the functions of AMs in the respiratory system (5). This mobility may also have an importance for the granulomatous response observed in the lung during *Mtb* infection (2).

Pro-inflammatory cytokines play crucial roles in the control of *Mtb* infection. IL-6 and TNF-α play important roles in the pathogenesis of TB, while IL-1α and IL-1β have been described as essential elements of the immune response against *Mtb* infection *in vivo*, able to coordinate the host defense for effective bacterial containment within the granuloma (27). In humans, decreased IL-1α in plasma has been correlated with active TB in contrast to patients with asymptomatic TB, indicating the importance of IL-1α in the control of *Mtb* infection and the relevance of this cytokine as a potential target for host-directed therapy (28). Furthermore, cells obtained by bronchoalveolar lavage from TB patients showed a strong spontaneous release of IL-1β, as well as IL-6 and TNF-α, mostly driven by AMs (29).

The anti-inflammatory cytokine IL-10 can decrease the host’s control over *Mtb* by impairing phagosome maturation and reducing the production of reactive oxygen species (30, 31). The negative influence of IL-10 on anti-mycobacterial defenses has been demonstrated in IL-10 deficient mice, for which *Mtb* infection resulted in a reduced bacterial load *in vivo* (32). The evaluation of the cytokine secretion profile of MPI cells after *Mtb* infection indicated a highly pro-inflammatory response, including a very strong secretion of IL-6, TNF-α, and IL-1α, as well as significant amounts of IL-1β. The innate response to live *Mtb* is induced partly *via* sensing of cell components of incoming mycobacterial particles *via* TLR2, TLR9, the C-type Lectin Receptors Mincle and Dectin-1, the scavenger receptor MARCO, and the cytoplasmic receptor NOD2 during the early phases of mycobacterial infection, while subsequent mycobacterial replication inside macrophages generates further cytokine production (33–35). Using HK *Mtb* particles, we previously observed the potent production of IL-6 and IL-1α induction but a lack of IL-10 induction in MPI cells (9, 10), findings that were also verified in this study. Furthermore, we demonstrate here the efficient production of TNF-α and the weak induction of IL-1β in HK *Mtb*-stimulated MPI cells. By contrast, infection of MPI cells with live mycobacteria resulted in a more potent induction of the pro-inflammatory cytokines tested. Using the same or a lower MOI of live compared to HK *Mtb*, we observed significantly higher levels of TNF-α, IL-1α, and IL-1β. In addition, all three cytokines were produced at earlier time points post-infection. Thus, our present data indicate that live *Mtb* induces a higher production of pro-inflammatory cytokines in MPI cells as compared to HK bacteria, probably because of its intracellular replication.

Acting on the same receptor, both IL-1α and IL-1β play crucial roles in anti-mycobacterial defense. Interestingly, we found more IL-1α secretion (up to 100 ng/mL 48 h post-infection) than IL-1β (590 pg/mL at the same time point, Figure 2). Mycobacterial gene expression modifies host macrophage responses through a functional ESX-1 secretion system, suppressing inflammasome activation and consequent IL-1β responses to virulent strains, while inflammasome activation may not be required for IL-1α secretion (9, 35, 36). This could explain the lower induction levels of IL-1β and emphasize the importance of a potent, *Mtb*-triggered IL-1α production in macrophages. Interestingly and in contrast to LPS and HK *Mtb* stimuli, challenge with live mycobacteria led to a delayed but efficient production of IL-10 in MPI cells. Since AMs are the primary targets of *Mtb*, the observed production of this anti-inflammatory cytokine is likely to be advantageous for this pathogen. Therefore, it is likely that *Mtb* developed mechanisms enabling the induction of IL-10. Nevertheless, further work is necessary to clarify the molecular details of *Mtb*-induced IL-10 and IL-1α secretion mechanisms of AMs and MPI cells.

Together, our results demonstrate that the MPI cell system represents a physiologically relevant model of *Mtb*-induced innate responses. This can be of primary importance for the study of the induction of both pro- and anti-inflammatory cytokines in TB, as well as for the establishment of functional host-directed therapies targeting the pathways induced by these cytokines. MPI cells also offer the possibility to study *Mtb* pathogenesis in a relevant, primary AM model. As MPI cells are continuously growing cells, which are easy to obtain in large quantities, the delineation of relevant cellular mechanisms of *Mtb* pathogenesis can be done using this system.

Other experiments are ongoing to further investigate the interactions between *Mtb* and this new model of host macrophages. In particular, a comparison of the transcriptional responses of BMDM, MPI cells, and AMs in response to *Mtb* infection will be conducted, as well as an investigation of the recognition and phagocytosis mechanisms. MPI cells can be efficiently transfected and gene knockout MPI cell lines relevant for *Mtb* pathogenesis can be made (9, 10), allowing genetic studies to be conducted using these cells and paving the way toward a better understanding of AM functions in TB pathogenesis. What is already clear based on the results we provide here is that having such self-renewable, primary cells at disposal is a strong benefit for experiments where primary cells are required. Indeed, the need for animal sacrifice to recover or generate macrophages is greatly reduced, as all the preliminary testing and optimization of experiments with AMs can be done using the MPI cells. While more remains to be done, this work highlights the usefulness of these innovative macrophages in TB research as well as in the more widespread field of lung diseases.

## Ethics Statement

Experiments involving pathogenic bacteria were performed in a biosafety level 3 laboratory (registration number KCDC-09-3-03) and all experimental protocols were approved by the Institutional Biosafety Committee of Institut Pasteur Korea. All animal experiments were approved by the Institutional Animal Care and Use Committee (IACUC) of Institut Pasteur Korea.

## Author Contributions

MW maintained the MPI cells and participated in all assays described in the manuscript. DK and VD performed additional experiments with cell adhesion, replication, and lipid loading. MW, KHP, and CW performed and analyzed the cytokine quantification experiments. VD and GF analyzed the results and wrote the manuscript.

## Conflict of Interest Statement

The authors declare that the research was conducted in the absence of any commercial or financial relationships that could be construed as a potential conflict of interest.
