# Supplementary material for: Mycobacterium tuberculosis Infection and Innate Responses in a New Model of Lung Alveolar Macrophages
Source: Front Immunol. 2018 Mar 12;9:438. doi: 10.3389/fimmu.2018.00438 (PMC5858468; doi:10.3389/fimmu.2018.00438)
Supplement: Supplementary file 1 [file Data_Sheet_1.PDF]

Mycobacterium tuberculosis infection and innate responses in a new model of lung alveolar macrophages

Minjeong Woo[1], Connor Wood[2], Doyoon Kwon[1], Kyu-Ho Paul Park[3], György Fejer[2]# and Vincent Delorme[1]#

1. Tuberculosis Research Laboratory, Institut Pasteur Korea, Seongnam, Gyeonggi, 13488 Republic of Korea

2. School of Biomedical & Healthcare Sciences, Peninsula Schools of Medicine and Dentistry, Plymouth University, Plymouth, UK

3. Applied Molecular Virology, Institut Pasteur Korea, Seongnam, Gyeonggi, 13488 Republic of Korea

# Address correspondence to: gyorgy.fejer@plymouth.ac.uk, vincent.delorme@ip-korea.org.

Supplementary materials

Supplementary Figures

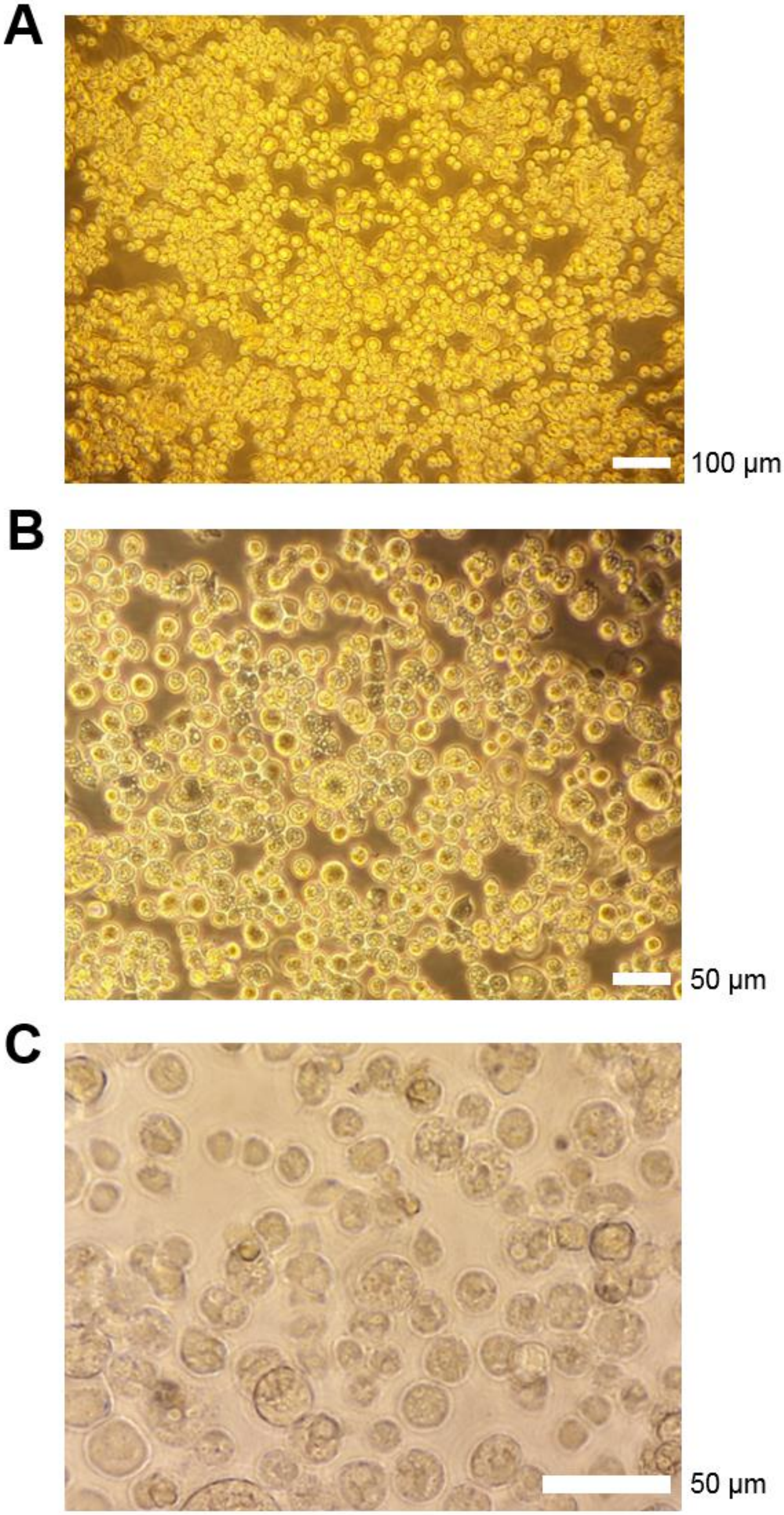

Figure S1. Observation of MPI cells (P16) at **A**. 10×, **B**. 20× and **C**. 40× magnification using light microscopy.

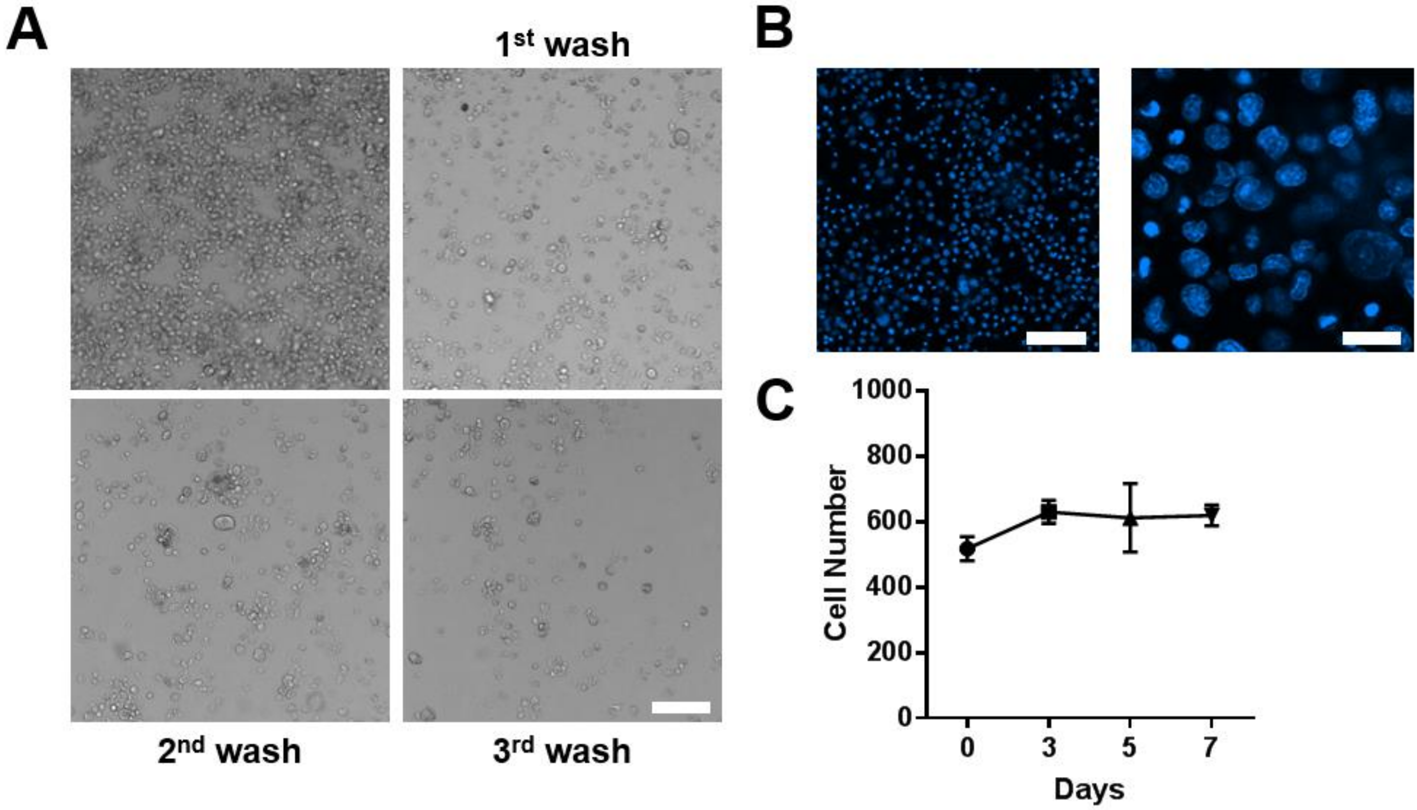

Figure S2. **A**. Bright-field images of live MPI cells (P18) plated at $5×10^5$ cells/mL in 384-well plates (50 µL/well) and residual attached cells after one, two or three washing using RPMI-FBS. Scale bar: 100 µm. **B**. MPI cells (P50) plated at similar densities, fixed with formalin and stained with Hoechst 33342, using a 2 min centrifugation step at 300 × *g* before each media change. Cells are shown at low (left) and high (right) magnification. Scale bar: left, 80 µm; right, 20 µm. **C**. Kinetic of replication of MPI cells (P60) after plating at $5×10^5$ cells/mL in 384-well plates without GM-CSF. Values are mean ± SD (n=15) for a representative experiment.

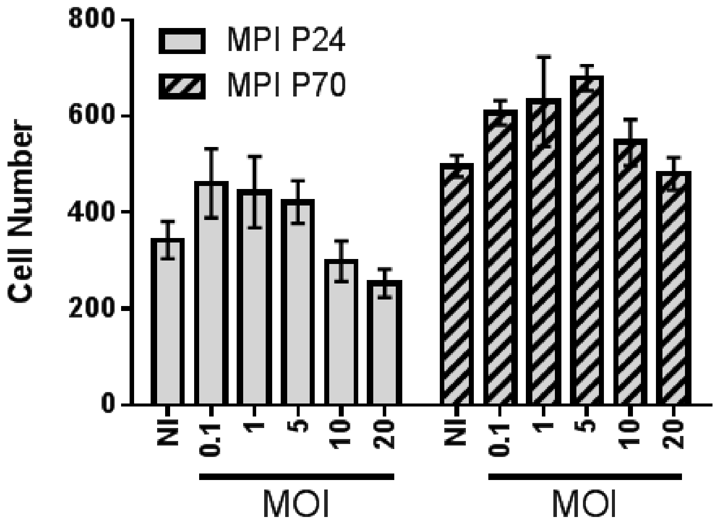

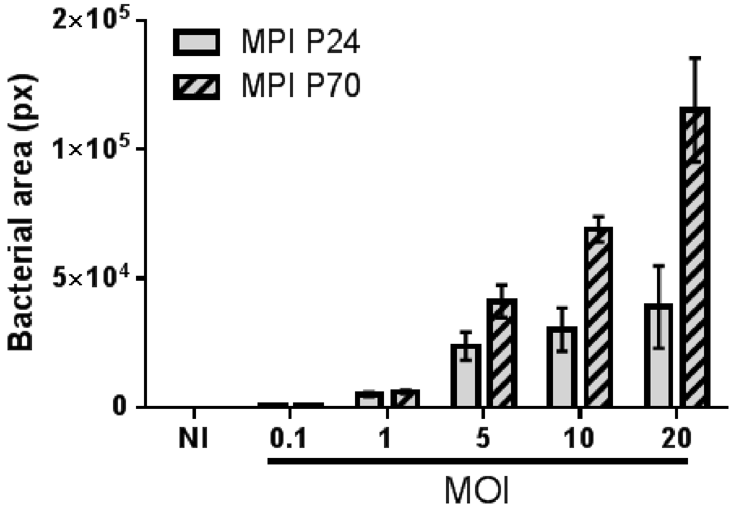

Figure S3. Quantification of the number of cells (top) and the total surface of bacteria (bottom) corresponding to the experiment shown in **Figure 1A**. Values are mean ± SD (n=4) for a representative infection experiment.

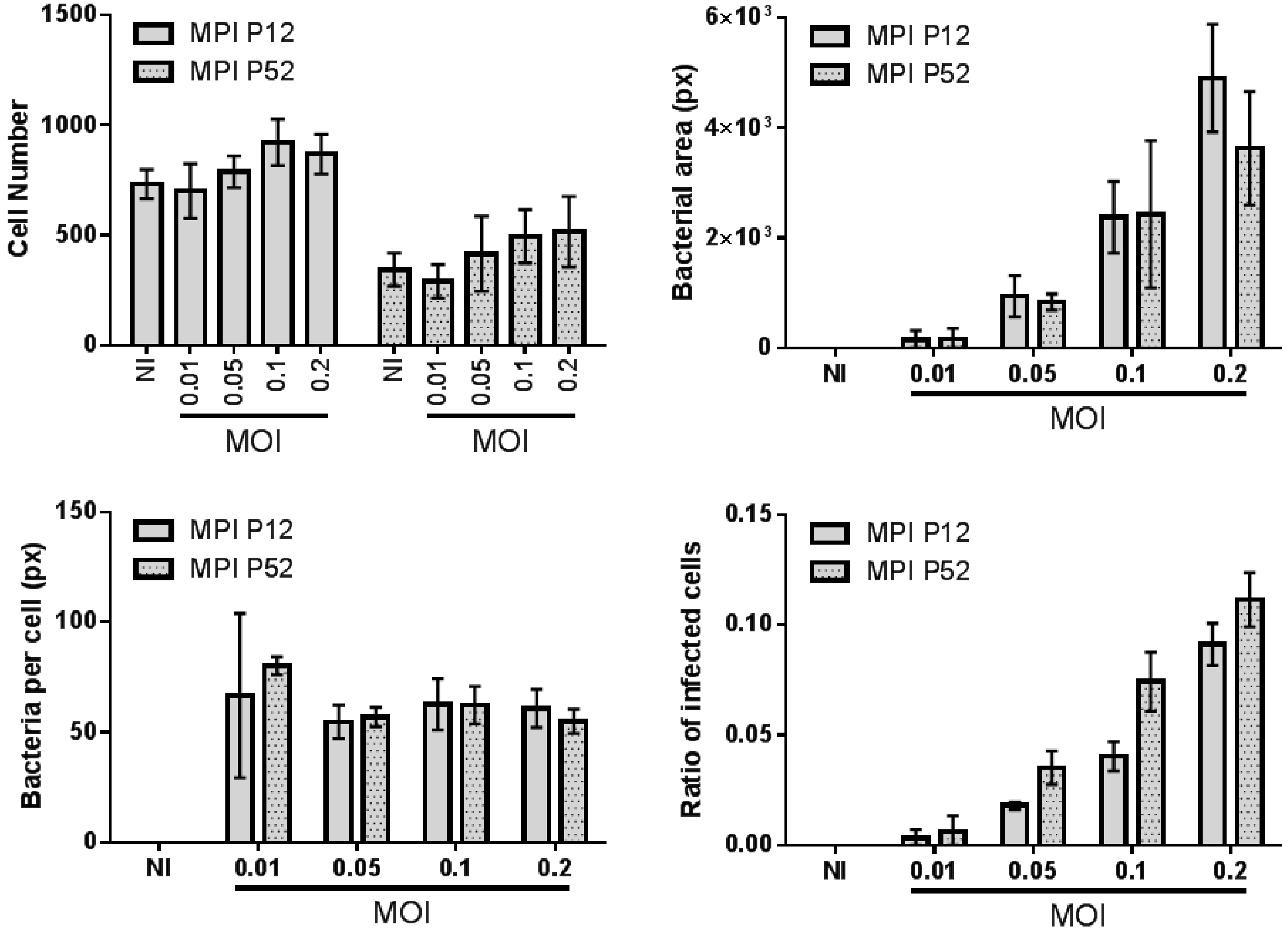

Figure S4. Comparison between MPI cells at passages 12 (P12) and 52 (P52), using *M. smegmatis*-GFP strain. Cells were plated and infected at different multiplicity of infection (MOI) for 4 h. Cells were washed with fresh RPMI-FBS, stained with Hoechst 33342 (5 µM final concentration) and imaged using fluorescence microscopy. Values are mean ± SD (n=4) for a representative infection experiment.

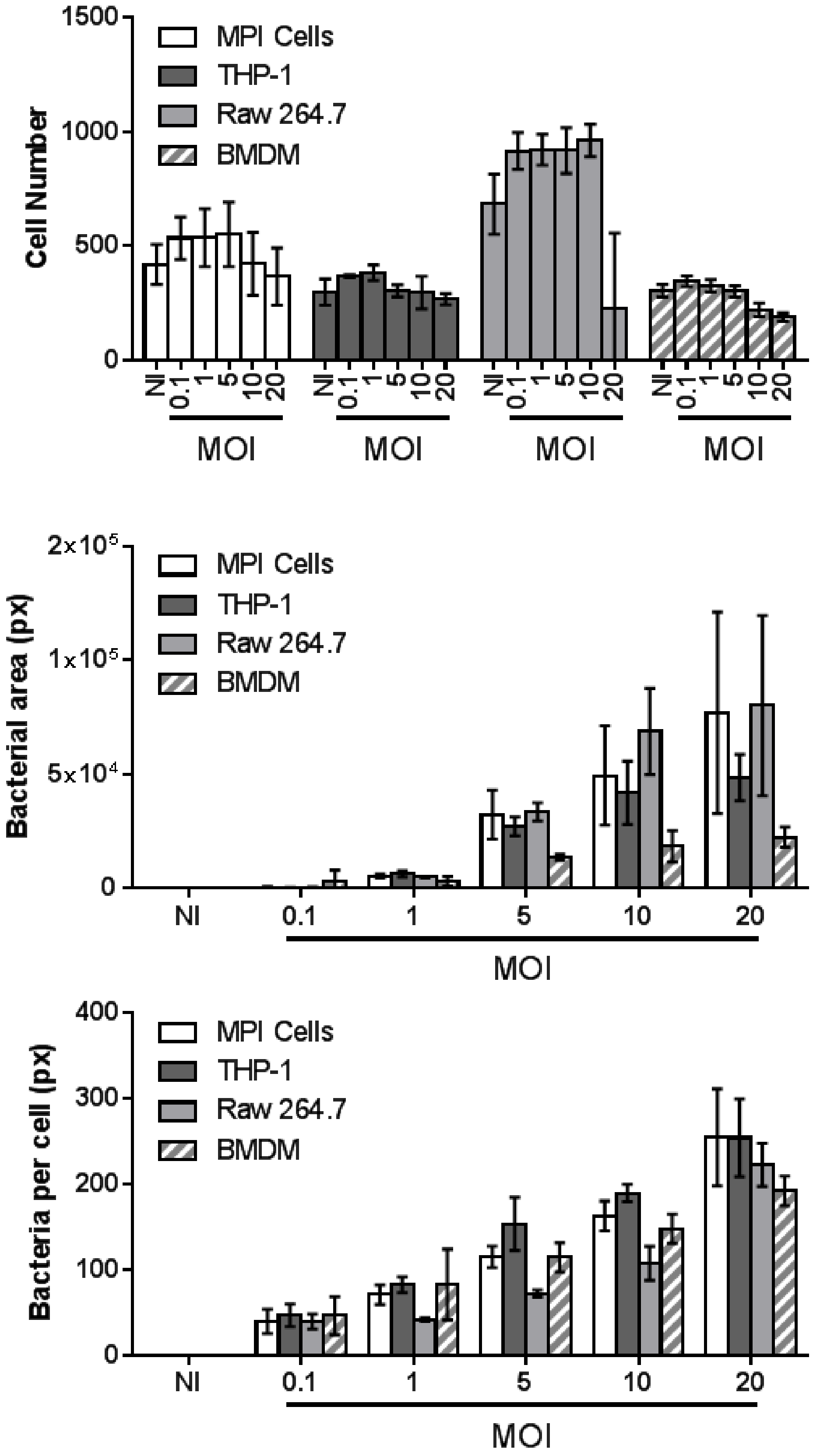

Figure S5. Quantification of the number of cells (top), the total surface of bacteria (middle) and the average area of bacteria per cell (bottom) corresponding to the experiment shown in **Figure 1B**. Values are mean ± SD (n=4) for a representative infection experiment.

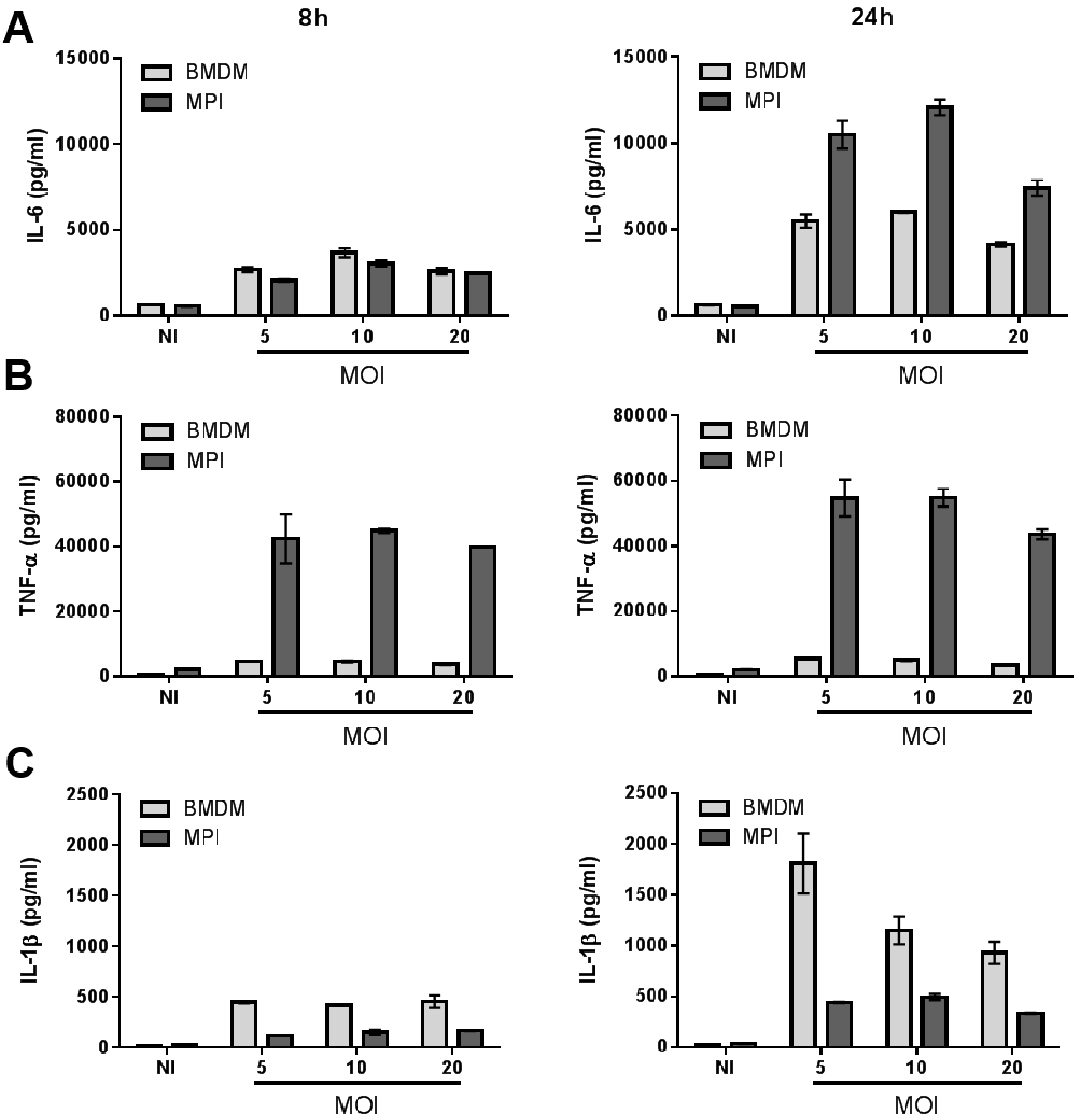

Figure S6. Comparison of the cytokine secretion profile between MPI cells (P22) and BMDM at 8h and 24h after infection with live *Mtb* at different MOI (5, 10 or 20). **A**. IL-6, **B**. TNF-α and **C**. IL-1β. Data are for a representative infection experiment and expressed as mean ± SD for 3 technical replicates.

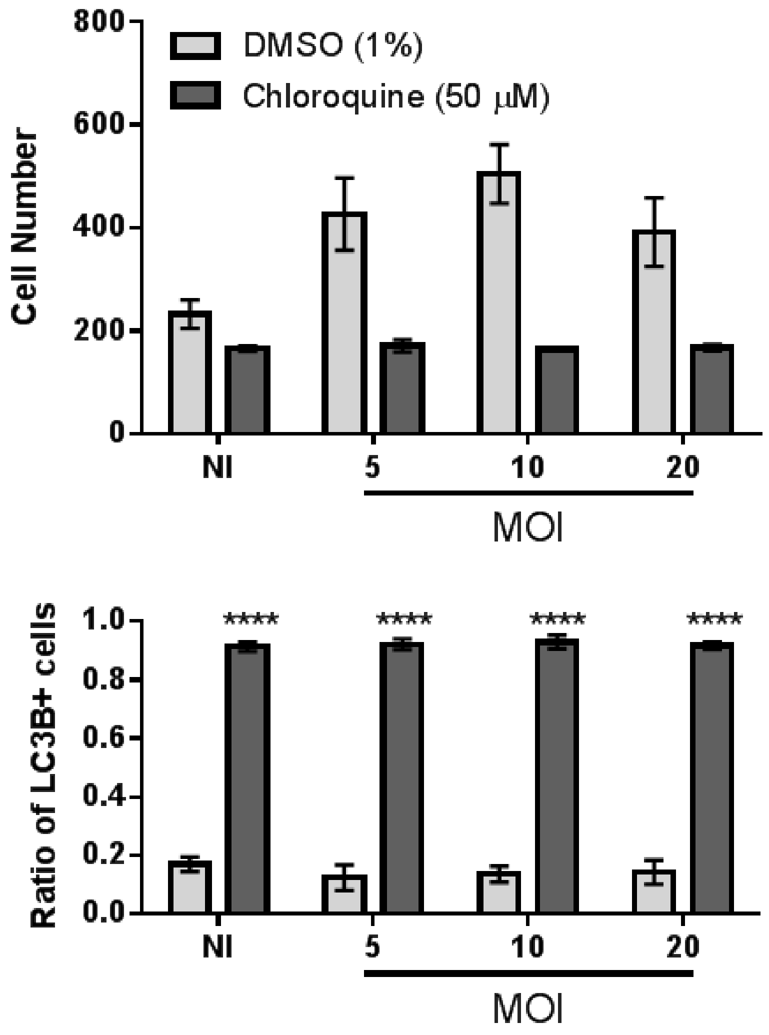

Figure S7. Quantification of the number of cells (top) and the ratio of cell displaying a LC3B positive signal (bottom), corresponding to the experiment shown in **Figure 4A-B**. Values are mean ± SD (n=3) for a single experiment. Significance between DMSO and chloroquine treated samples were calculated by Prism software using a two-way ANOVA with Tukey's multiple comparisons test. ****, p-value < 0.0001.

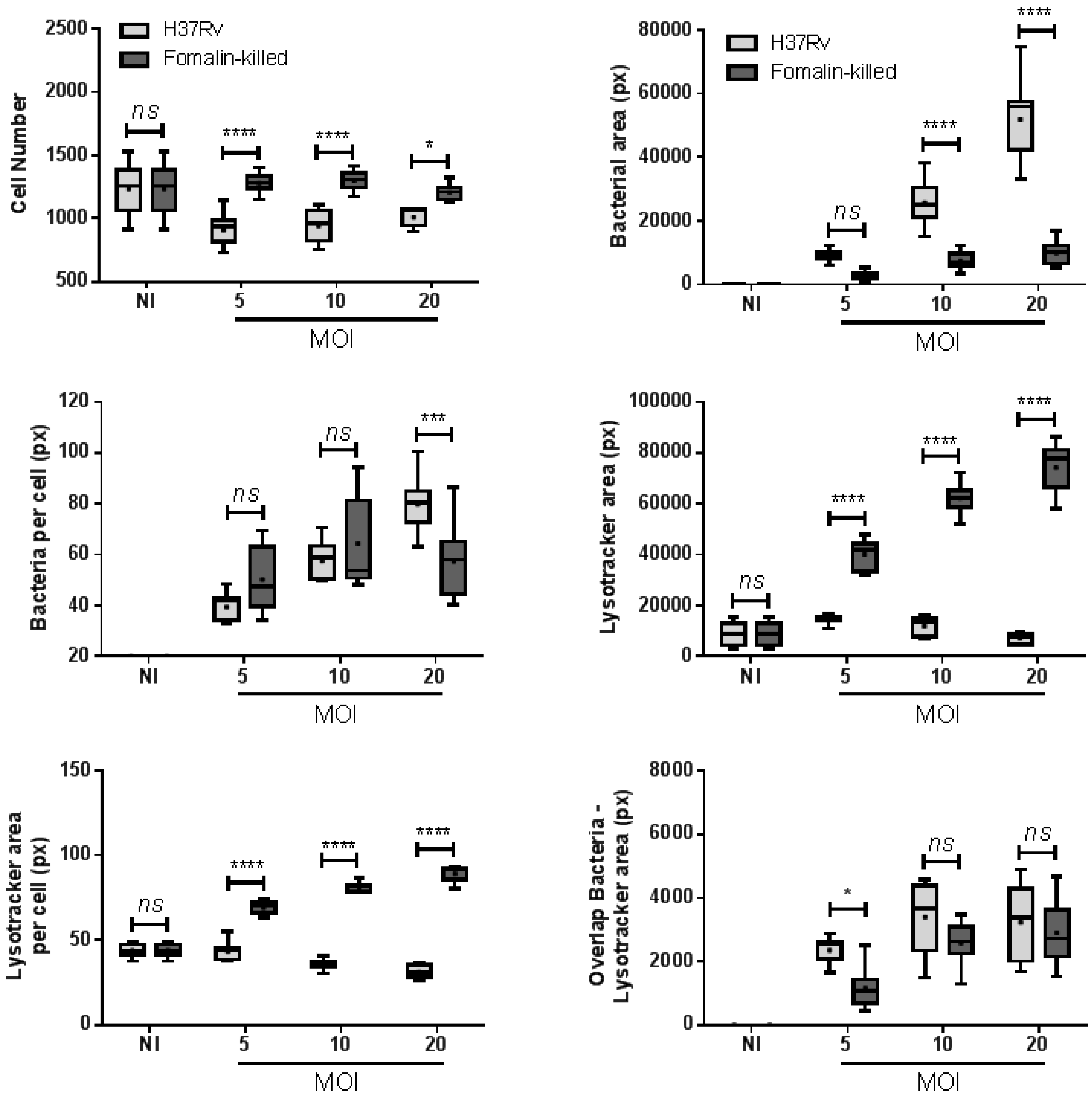

Figure S8. Quantification of the number of cells, bacterial area, bacteria per cell, LysoTracker area, LysoTracker area per cell and overlap area between bacteria and LysoTracker, corresponding to the experiment shown in **Figure 4C-D**. Values are mean ± SD (n=9) for a representative infection experiment. Significance between live and formalin-killed H37Rv bacteria were calculated by Prism software using a two-way ANOVA wirh Tukey's multiple comparisons test. *ns*, non-significant; *, p-value < 0.05; ***, p-value < 0.0005; ****, p-value < 0.0001.

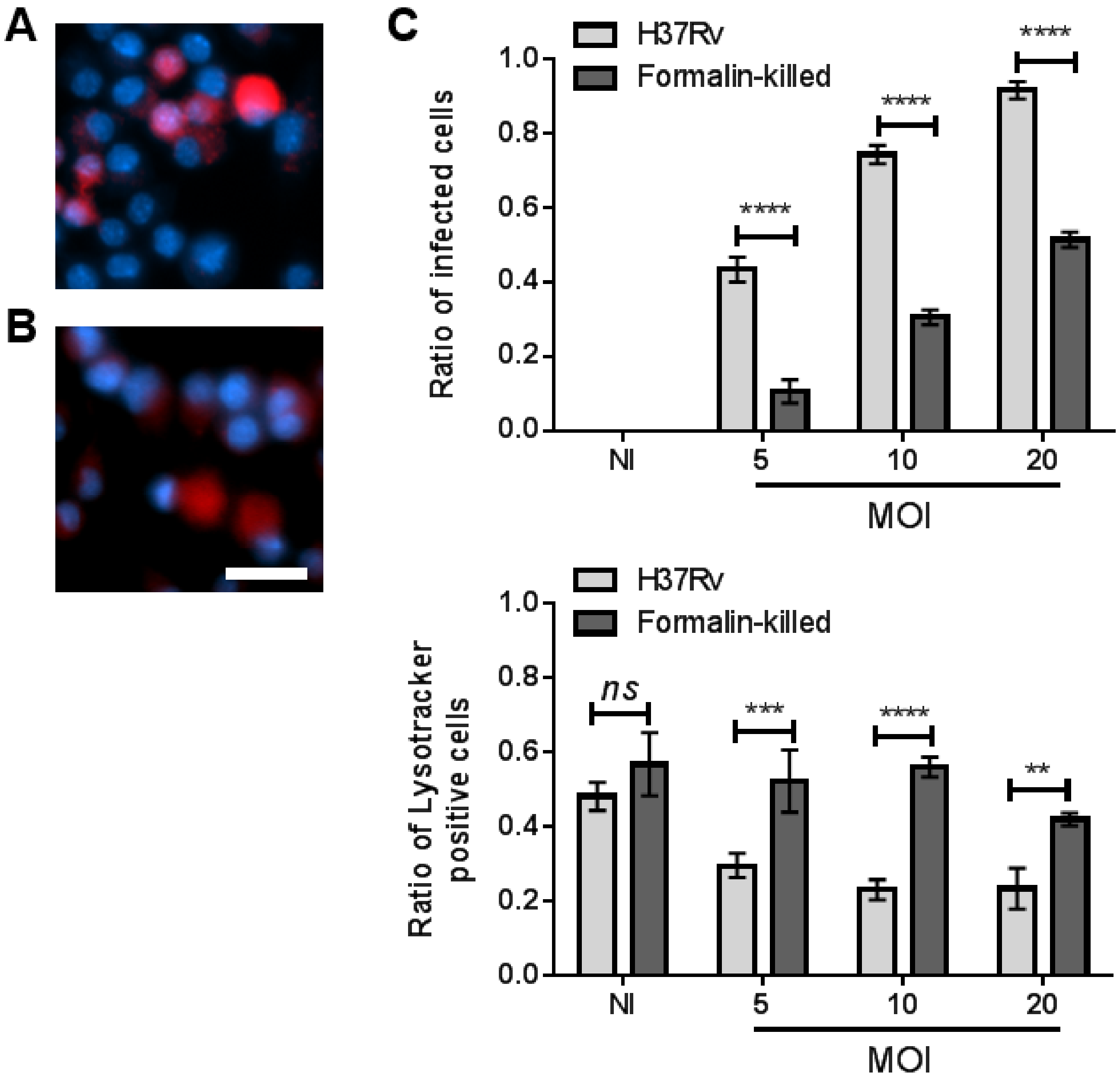

Figure S9. Non-specific background staining obtained for non-infected (**A**) Raw 264.7 cells and (**B**) THP-1 cells (blue) after staining with LysoTracker (red). The color visualization settings used here are the same as those used for **Figure 4C**. Scale bar: 25 µm. **C**. Typical result of LysoTracker staining experiment using Raw264.7 cells. Values are mean ± SD (n=6) for a single infection experiment. Significance between live and formalin-killed H37Rv bacteria were calculated by Prism software using a two-way ANOVA with Tukey's multiple comparisons test. *ns*, non-significant; **, p-value < 0.005; ***, p-value < 0.0005; ****, p-value < 0.0001.
